# Automated Artificial Intelligence-Based Assessment of Lower Limb Alignment Validated on Weight-Bearing Pre- and Postoperative Full-Leg Radiographs

**DOI:** 10.3390/diagnostics12112679

**Published:** 2022-11-03

**Authors:** Felix Erne, Priyanka Grover, Marcel Dreischarf, Marie K. Reumann, Dominik Saul, Tina Histing, Andreas K. Nüssler, Fabian Springer, Carolin Scholl

**Affiliations:** 1 Siegfried Weller Institute for Trauma Research, BG Unfallklinik Tübingen, Eberhard Karls University Tübingen, 72076 Tübingen, Germany; 2 Department for Traumatology and Reconstructive Surgery, BG Unfallklinik Tübingen, Eberhard Karls University Tübingen, 72076 Tübingen, Germany; 3 RAYLYTIC GmbH, 04109 Leipzig, Germany; 4 Robert and Arlene Kogod Center on Aging, Mayo Clinic, Rochester, MN 55905, USA; 5 Department of Radiology, BG Unfallklinik Tübingen, Eberhard Karls University Tübingen, 72076 Tübingen, Germany

**Keywords:** artificial intelligence, deep learning, lower limb alignment, automatic analysis, X-ray, full-leg

## Abstract

The assessment of the knee alignment using standing weight-bearing full-leg radiographs (FLR) is a standardized method. Determining the load-bearing axis of the leg requires time-consuming manual measurements. The aim of this study is to develop and validate a novel algorithm based on artificial intelligence (AI) for the automated assessment of lower limb alignment. In the first stage, a customized mask-RCNN model was trained to automatically detect and segment anatomical structures and implants in FLR. In the second stage, four region-specific neural network models (adaptations of UNet) were trained to automatically place anatomical landmarks. In the final stage, this information was used to automatically determine five key lower limb alignment angles. For the validation dataset, weight-bearing, antero-posterior FLR were captured preoperatively and 3 months postoperatively. Preoperative images were measured by the operating orthopedic surgeon and an independent physician. Postoperative images were measured by the second rater only. The final validation dataset consisted of 95 preoperative and 105 postoperative FLR. The detection rate for the different angles ranged between 92.4% and 98.9%. Human vs. human inter-(ICCs: 0.85–0.99) and intra-rater (ICCs: 0.95–1.0) reliability analysis achieved significant agreement. The ICC-values of human vs. AI inter-rater reliability analysis ranged between 0.8 and 1.0 preoperatively and between 0.83 and 0.99 postoperatively (all *p* < 0.001). An independent and external validation of the proposed algorithm on pre- and postoperative FLR, with excellent reliability for human measurements, could be demonstrated. Hence, the algorithm might allow for the objective and time saving analysis of large datasets and support physicians in daily routine.

## 1. Introduction

Malalignment of the leg axis can be congenital or acquired. Physiological bone morphology of the knee changes over a lifetime, for example, due to physical activity in youth, sports trauma, or osteoarthritis in old age [1,2,3,4]. The assessment of the knee alignment using standing full-leg radiographs (FLR) is an established and standardized method. This “vital imaging modality” of weight-bearing radiographs allows for the determination of the load-bearing axis of the leg [5]. The determination of the load-bearing axis represents indispensable information for diagnostic and individual therapy planning. Especially when performed in full extension and with appropriate rotation, the application of FLR is optimal and comprehensible [6]. Nevertheless, manual measurements on these radiographs are time-consuming and laborious. Due to differences in the definition of the joint lines, experience of different examiners, or even the incentive to adhere to quality requirements, there is a large risk of bias in measuring knee alignment angles [7,8,9]. Furthermore, the objective of many therapeutic approaches is the correction of symptomatic malalignment. Thus, there is a great need for reliable, independent, and objective measurements for proper medical decision-making.

Research efforts for an automatic solution to measure knee alignment angles on FLR are constantly ongoing. In 2007, Goker et al. presented a novel semi-automated digital method using common image software for the designation of landmarks and determination of angles [10]. In 2010, Fakhrai et al. developed a software solution for the automatic assessment of the knee alignment angles, in addition to an observational study including a small cohort of 15 patients with native knee joints [11]. The interest in finding an automatic solution increased with the exponentially growing computing power and technological advances in the field of artificial intelligence (AI). Thus, the number of published scientific articles about AI has grown significantly in the last three years [12]. However, only a few research groups have published findings about the automatic assessment of the knee alignment angles on FLR, and only limited external validation exists in the current literature [11,13,14,15,16]. In particular, the set of evaluated radiographic parameters is small or the separate evaluation of the performance of the algorithms on preoperative and postoperative images is missing altogether. The generalizability of the algorithm and precise measurement of lower limb alignment are essential for individual treatment planning in clinical routine, national quality control activities, the generation of medical guidelines, internal and external quality management, and the maintenance of register databases [17,18,19,20].

The aim of this study is to develop a new AI-based algorithm to automatically determine lower limb alignment angles in weight-bearing FLR. The study also provides an independent and thorough validation of the algorithm for five radiographic parameters by comparing its measurements to human expert measurements. By distinguishing between the preoperative and postoperative performance, the quality of the algorithm is evaluated in different challenging scenarios. 

## 2. Materials and Methods

### 2.1. Patient Selection and Sample Description

The study was approved by the ethics committee Tübingen, Germany (ID: 197/2021BO2). The validation dataset was recruited from a cohort of 119 patients having undergone total knee arthroplasty (TKA). According to the standardized approach, weight-bearing, antero-posterior FLR were taken at least 3 months preoperatively and 3 months postoperatively. All available FLR were anonymized and transferred to a database. In this context, the term preoperative FLR refers to a native knee, in the absence of a knee implant, and the term postoperative FLR refers to the presence of a TKA implant. Images with other knee implants, such as osteosynthesis plate, unicondylar knee arthroplasty, or distal femur replacement, were excluded. Invalid images caused by inaccurate acquisition techniques were excluded (e.g., truncated/incomplete images by misalignment or overexposure of images by severe obesity with hanging bulge of skin and fat tissue from the lower abdomen). Both the pre- and postoperative datasets contained images with hip implants. 

### 2.2. Validation Images and Measured Parameters

Weight-bearing antero-posterior full-leg X-rays were taken in three parts and stitched together in a standardized process using software from a digital X-ray system by Philips Healthcare (Philips GmbH Market DACH, Hamburg, Germany). The storage format was DICOM (digital imaging and communications in medicine). The following angles were measured on the anonymized images:

- mFAmTA: angle between the mechanical axis of the femur and the mechanical axis of the tibia, also known as the hip-knee-ankle angle.
- FSAmTA: angle between the anatomical femur shaft axis and the mechanical axis of the tibia.
- mMPTA: angle between the mechanical axis of the tibia and the tibial plateau knee joint line, measured on the medial side.
- mLDFA: angle between the mechanical axis of the femur and the femoral condyles knee joint line, measured on the lateral side.
- mLDTA: angle between the mechanical axis of the tibia and the tibial plafond, measured on the lateral side.

All angles were measured in degrees. The validation dataset contained both bilateral and single leg X-rays.

### 2.3. Manual Measurement Procedure

Manual measurements were performed using the software suite MediCAD^®^ 2D classic (mediCAD Hectec GmbH, Altdorf/Landshut, Germany). The software allows adjustment for brightness and contrast, as well as magnification. To assess inter-rater reliability, all preoperative images were measured by the orthopedic surgeon (rater 1), and a second physician trained and experienced in radiographic measurements (rater 2). Postoperative images were measured only by rater 2. Rater 2 conducted all pre- and postoperative measurements twice, at two different time points, to determine intra-rater reliability (rater 2a and 2b).

### 2.4. Automated AI-Workflow and Training Procedure

**Figure 1:**
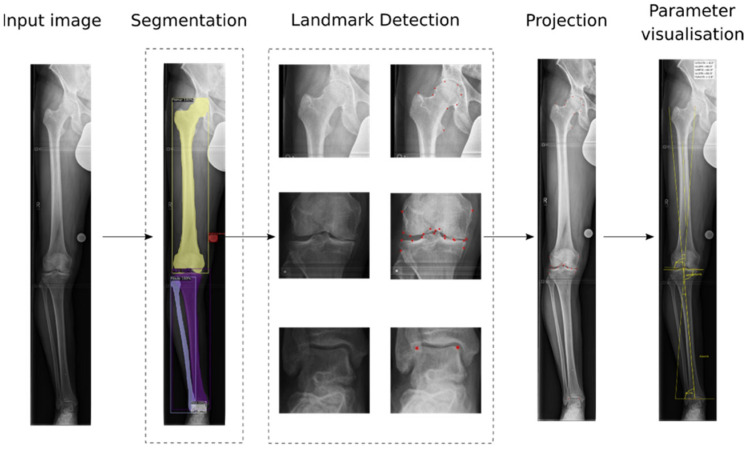
Fully automatic AI algorithm for the determination of lower limb alignment angles. Based on the predicted segmentation masks around anatomical structures and implants, crops of the proximal femur, knee area, and ankle joint were created. Landmark detection models predicted landmarks on these smaller crops. The landmarks were projected back on the original image, and the final parameters were computed and visualized.

A fully automatic AI-based algorithm was developed for the determination of the lower limb alignment angles. The algorithm works in multiple stages, and the workflow is illustrated in Figure 1. It consists of five deep convolutional neural networks (CNNs) and is triggered with the input of a full-leg X-ray. After passing through all stages, the measured angles are visualized. For bilateral images, all parameters are computed for both legs separately. 

The training datasets are described per model below. The images used for training the models were completely independent of the validation images.

### 2.5. Preprocessing

Each DICOM image was anonymized and normalized with the use of the window width and window center information from the DICOM tags. 

### 2.6. Segmentation of Anatomical Structures and Implants

After preprocessing, a segmentation model was used to automatically detect and segment all anatomical structures and implants in the image. Polygon masks and rectangular boxes around the bones and implants were predicted (see Figure 1). 

#### Training of the Segmentation Model

To train the segmentation model, a dataset of training images was manually annotated with bounding polygon masks by trained medical staff. The regions of interest included femur, fibula, tibia, talus, and implants. The training dataset consisted of 202 unilateral antero-posterior FLR (192 preoperative, 10 postoperative) from two independent clinical sites. Horizontal flipping was applied to half of the training set, for the purpose of data augmentation, resulting in a trained model that is insensitive to the leg side. The instance segmentation model was based on mask-RCNN architecture, implemented in the Pytorch framework [21,22]. Pre-trained weights from https://pytorch.org/serve/model_zoo.html (accessed on 23 June 2021) were used to initialize the model. Training ran for 200 epochs on a NVIDIA GeForce GTX 1080 GPU, with a learning rate of 0.002.

### 2.7. Landmark Placement

In the next step, the bounding boxes determined by the segmentation model were used to generate smaller crops for the landmark detection models. The leg side was determined based on the relative position of fibula and tibia. The images were flipped accordingly to train the landmark placement models on the right leg crops. 

#### Training of the Landmark Placement Models

In total, four different landmark detection models were trained: one for the proximal femur, including the greater and lesser trochanter, as well as the femoral head (9 landmarks), one for a TKA implant (15 landmarks), one for the preoperative knee joint (20 landmarks), and one for the talus (2 landmarks). Depending on the detection of a TKA implant, the appropriate model for the knee area was triggered. Contrast enhancement was applied to the crops to increase visibility of structures. 

To train the proximal femur landmark detection model, a crop was made by taking the upper quarter of the femur bounding box. Landmarks were placed on the femoral head, femoral neck, and the greater and lesser trochanter by trained medical staff. The training set consisted of 326 images (320 preoperative, 6 with hip implants) from two clinical sites.

The TKA landmark detection model was trained on crops based on the bounding box around the femoral and tibial parts of the TKA implant. Five landmarks were placed on the femoral part, and ten landmarks were placed on the tibial part of the implant on pre-defined locations optimal for determination of parameters. Trained medical staff manually placed these landmarks on a total of 190 images with TKA implants from two clinical sites. 

The crops for the knee landmark detection models were generated by merging the lower eighth of the femur and the upper eighth of the tibia bounding boxes detected by the segmentation model. In total, 20 landmarks were placed in pre-defined positions on the femoral condyles and tibial plateau. The training set consisted of 319 images (288 preoperative, 30 images with unicondylar implants) from two clinical sites. 

For the training of the talus landmark detection model, the predicted talus bounding boxes were directly used to create the crops. Two landmarks were placed on the superior articular surface of the talus. In total, 320 images from two sites were used for training. 

Manual landmark placement by the trained medical staff was conducted in a custom graphical user interface (GUI) developed in Python. The landmarks were constantly reviewed for quality and consistency. During training of each model, data augmentation was applied by rotating and/or scaling the crops by random factors. The crops were then downscaled to 256 × 256 pixels. The network was adapted from UNet and implemented in TensorFlow [23,24]. Training of all models ran on a NVIDIA GeForce GTX 1080 GPU, with a constant learning rate of 0.001 for 100 epochs each.

### 2.8. Projection and Parameter Computation

In the final two stages, the coordinates of the predicted landmarks on the crops were projected back to the original image. The segmentation masks and projected landmarks were then used to compute the five lower limb alignment parameters. In case of prediction failures (e.g., the talus was not detected by the segmentation model), the affected parameters were not computed. The remaining parameters, however, were still determined, resulting in individual detection rates per parameter.

### 2.9. Statistical Analysis

For preoperative images, intra- (R2a vs. R2b) and inter-rater analyses (R1 vs. R2a, R1 vs. R2b, and each rater against the algorithm R-AI) were conducted. Only intra-rater analysis was performed for postoperative images. Agreement within and between raters was quantified by mean differences (95% confidence interval (CI), standard deviation (SD)), root mean square error (RMSE), Pearson’s correlation coefficient r, and single-measure intra-class correlation coefficients (ICC) for absolute agreement. Python 3 programming language was used for all statistical computations.

## 3. Results

### 3.1. Patient Sample

The initial cohort consisted of 119 patients, with age span of 44 to 85 years (mean 66 ± 9.3) and gender distribution of 39% male and 61% female. After application of exclusion criteria involving the availability of ground truth values for all parameters, the final dataset consisted of 95 preoperative and 105 postoperative weight-bearing, antero-posterior FLR. Some of the patients were undergoing a second surgery and had TKA on one of the knees operated on in a previous timepoint. In such cases, and if bilateral images were present, parameters were measured on both TKA implants in the postoperative image, but only the native knee was measured in the preoperative image. Therefore, the number of postoperative FLR was higher than preoperative FLR.

### 3.2. Human vs. Human Intra-Rater Reliability Analysis

Table 1 displays the results of the intra-rater reliability analysis, based on the repeated measurements of all pre- and postoperative images by rater 2. The intra-rater agreement, as defined by the ICC-value, was highest for mFAmTA (ICC_preop_ = ICC_postop_ = 1.0) and lowest for mLDFA (ICC_preop_ = 0.95, ICC_postop_ = 0.97). All comparisons reached significant agreement (all *p* < 0.001). The RMSE were smallest for mFAmTA (0.3° for preoperative, 0.3° for postoperative images) and largest for mLDTA (1.2° for preoperative, 1.3° for postoperative images). 

### 3.3. Human vs. Human Inter-Rater Reliability Analysis

The preoperative images were measured by two raters to allow for an inter-rater reliability analysis. The results are displayed in Table 2. The agreement between raters was highest for the two parameters mFAmTA and FSAmTA (ICC_preop_ = 0.99 for both R1 vs. R2a and R1 vs. R2b). Again, the agreement was significant (*p* < 0.001) for all comparisons. The agreement was lowest for mLDTA (ICC_preop_ = 0.88 for R1 vs. R2a, ICC_preop_ = 0.85 for R1 vs. R2b). The RMSE were largest for mMPTA (1.6° for R1 vs. R2a, 1.7° for R1 vs. R2b) and smallest for FSAmTA (1.0° for R1 vs. R2a, 1.1° for R1 vs. R2b).

### 3.4. AI Detection Rates

For preoperative images, mMPTA, mLDFA, and FSAmTA could be computed in 98.9% of cases. mLDFA and mFAmTA could be determined in 92.6% of cases. For postoperative images, mMPTA and mLDTA were computed in 97.1% of cases. The detection rates for postoperative FSAmTA, mLDFA, and mFAmTA were 95.2%, 94.3%, and 92.4%, respectively.

### 3.5. Human vs. AI Inter-Rater Reliability Analysis

**Figure 2:**
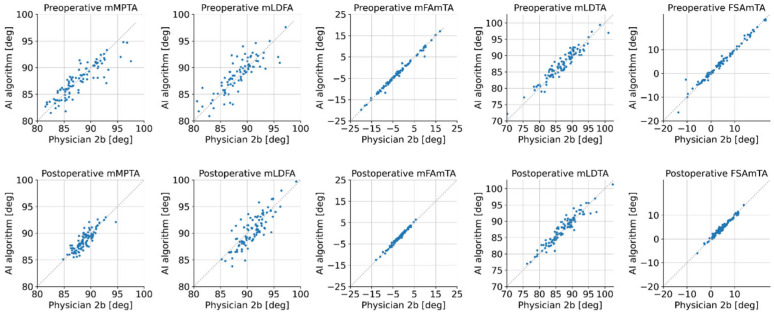
Correlations between the manual measurements of rater 2b on the x-axes and the automatic measurements by the AI algorithm on the y-axes, respectively.

The agreement between the automatic measurements and human raters was largest for the two parameters mFAmTA and FSAmTA (exemplarily: ICC_preop_ = ICC_postop_ = 0.99 for R-AI vs. R2b) and smallest for mLDFA (ICC_preop_ = 0.83, ICC_postop_ = 0.85 for R-AI vs. R2b). The correlations between measurements of the AI algorithm and rater 2b are visualized in Figure 2. When considering all comparisons, the ICC-values ranged between 0.8 and 1.0 preoperatively and between 0.83 and 0.99 postoperatively (all *p* < 0.001). For preoperative images, the RMSE ranged between 1.6°–1.8° for mMPTA, 1.9°–2.2° for mLDFA, 0.7°–0.9° for mFAmTA, 1.5°–2.2° for mLDTA, and 1.1°–1.2° for FSAmTA. For postoperative images, the RMSE for the comparisons of automatic measurements against the two measurements by rater 2 were 1.0° and 1.0° for mMPTA, 1.6° and 1.7° for mLDFA, 0.5° and 0.5° for mFAmTA, 1.5° and 1.5° for mLDTA, and 0.5° and 0.6° for FSAmTA, respectively.

## 4. Discussion

### 4.1. Critical Comparison with Literature

Previous scientific publications in the field of AI for the automated assessment of lower limb alignment utilized different software architectures and approaches. The “YOLOv4 And Resnet Landmark regression Algorithm” (YARLA) by Tack et al. represented a first step toward a fully automated assessment of knee alignment [13]. The accuracy of hip-knee-ankle angle computations was assessed on 2943 radiographs by comparing the results of two independent, publicly accessible image analysis studies. The average deviation of manually placed landmarks and automatically detected ones was less than 2.0 ± 1.5 mm for all structures. The average mismatch between hip-knee-ankle angle determinations was 0.09 ± 0.63°, which is in the same error range as the algorithm presented here (mean error R2a vs. AI: preoperative: 0.0°, postoperative: 0.15°). Compared to our algorithm, the method proposed by Tack et al. demonstrated a slightly higher detection rate of 99.85% for the hip-knee-ankle, which may be due to the larger training set of 900 images. Nevertheless, our approach achieved remarkable detection rates, with approximately one third of the amount of training data. Due to differences in the validation images, comparing the performance of the two methods is challenging. Nevertheless, the reported ICCs for the hip-knee-ankle angle were similar (0.98 by Tack et al. vs. 0.99 presented in this study). Our proposed method can determine four additional parameters apart from the hip-knee-ankle angle, which was the only parameter considered by Tack et al. Furthermore, in the presented study, there was a clear distinction between pre- and postoperative analyses, and the similarity in their statistical evaluation indicates the robustness and generalizability of our algorithm.

Notably, the results for FSAmTA and the hip-knee-ankle angle (mFAmTA) were generally better than for angles between the knee joint lines and the long axes of the bones. This applies to both the comparisons between two sets of human measurements and comparisons between human and AI measurements (see Table 1, Table 2 and Table 3). However, all ICC-values are still considered excellent, indicating that an accurate automatic determination is possible, even for these more challenging parameters.

Simon et al. trained an algorithm on over 15,000 radiographs to measure various clinical angles and lengths from standing long-leg radiographs [14]. AI and expert measurements were performed independently. A total of 295 long leg radiographs from 284 patients were analyzed. The AI model produced outputs on 98.0% of the images. AI vs. mean observer revealed mean-absolute-deviation between 0.39° and 2.19° for angles and 1.45–5.00 mm for lengths. Similar to the results presented here, their algorithm demonstrated excellent reliability in all lengths and angles (ICC ≥ 0.87). Again, no differentiation between preoperative and postoperative measurements were reported, so a scientific comparison was not possible within the scope of this study. The authors profess an algorithm with reproducible, accurate measures and time savings. On average, their algorithm was 130 s faster than the clinicians [14]. Compared to our novel algorithm, they exhibited similar overall reliability and accuracy. The calculation of the time savings was not part of our study. In our opinion, a determined calculation was very subjective and depended on computing power. To provide a preliminary time value, using hospital’s medical informatics infrastructure, our new algorithm can process a fully automated assessment of lower limb alignment in less than 60 s. 

What all above-mentioned solutions have in common is a lack of transparency into the black-box mechanism of the machine learning algorithm. We are convinced that the serial combination of five consecutive algorithms and intermediate visualization of predicted segmentations and landmarks is suitable to disclose the localization and the exact reason for the potential errors. Our final software solution would include GUI control for the visualization of the single steps. This is the first step toward a warning mechanism for borderline cases.

### 4.2. General Limitations of Method

The basic procedure of measuring lower limb alignment remains unchanged in the presented method. The use of AI may result in time saving, fatigue-free controls, objectified examination procedures, and easy investigation in large volumes of images. However, fundamental procedural deficiencies persist, as a result of the inherent technique of weight-bearing FLR. Jud et al. measured the hip-knee-ankle angle in simulated antero-posterior FLR. Deviations caused by rotation up to 30°, flexion up to 30°, or varus/valgus up to 9° did not vary more than 3° from median values. Their findings concluded that deviations in hip-knee-ankle angle measurements are comparable in patients with different coronal alignment [6]. However, Brouwer et al. pointed out several pitfalls in determining knee alignment in a cadaver study, e.g., simultaneous flexion of the knee and rotation of the leg induced large changes in projected angles. However, flexion of the knee without rotation of the lower extremity had little effect on angles, as projected on full-length ante-posterior radiographs. Similarly, the rotation of the lower extremity without flexion of the knee also had little effect [25]. Zahn et al. showed that the postoperative mechanical axis correlates with limb loading. They used two digital scales separately to capture the load of each limb during X-ray imaging. The mechanical axis changed from an initial ten days after surgery −1° ± 2° valgus alignment to three months after surgery varus axis of +1° ± 2°. The alterations were much more pronounced in patients with postoperative incomplete extension [26]. In alignment with Zahn et al. and the authors mentioned above, an interval of three month prior to surgery appears to be sufficient. The algorithm itself does not address the specific structural measurement inaccuracy of the technique, and future warning mechanisms, regarding malrotation and contractures, may be useful.

### 4.3. Limitations of the Study

The size of the validation dataset was limited. However, our database increases automatically with time, allowing for the continuous improvement of the algorithm. Furthermore, the automated assessment of lower limb alignment was only evaluated on native knees and patients with TKA, even though the field of clinical application was larger, due to the variety of available implants. Therefore, we plan to include other implant types like plates or unicompartmental knee arthroplasty in the validation dataset. The postoperative images were only measured by rater 2, preventing an inter-rater reliability analysis between the two human raters. However, based on the excellent inter-rater reliability for pre-operative images, a comparable agreement between the two raters would be expected for postoperative images, too. Future studies, including multiple raters, may prove this assumption. 

### 4.4. Outlook and Connecting Factors

There are several possible extensions to our algorithm. The measurements of alignment angles on different types of protheses, such as unicompartmental knee arthroplasty, could be the next step. Other parameters, such as the joint line orientation angle, described as the angle between the knee joint line and the floor, could be assessed; this parameter was associated with worse postoperative outcomes in unicompartmental knee arthroplasty [27]. Leg length discrepancies are not commonly associated with TKA, but large changes in the leg length are common after hinged TKA. Labott et al. reported an absolute mean and median change in leg lengths of 20 mm and 13 mm [28]. Those measurements could be integrated into the existing algorithm.

In the future, a holistic set of algorithms is conceivable by combining the achievements of several research teams. For example, a supplementary automated “Sarcopenia Screening”, as well as an additional algorithm for the identification of specific orthopedic implant models from imaging, could be combined [29,30]. Another approach could be the integration of clinical factors, such as lab results or quality of life questionnaires. Bonakdari et al., for example, developed a comprehensive machine learning model that bridges major osteoarthritis risk factors, serum levels of adipokines, and related inflammatory factors to predict the risk of disease [31]. 

Recent studies point out a more differentiated consideration of the knee joints’ physiology. Subtypes of different physiological characteristics, such as constitutional varus knees, have been described [32]. The performance of TKA changed from “bone surgery” to “soft tissue surgery”. New concepts of kinematic alignment are paving the way for individual treatment options [33]. In the future, the accurate and fully automated algorithm for assessment of knee alignment can replace arduous routine tasks, leaving more time for careful diagnosis and the selection of appropriate treatment options. For instance, the algorithm could be automatically triggered after an X-ray is taken in the hospital. The clinician could then review and potentially correct or immediately approve the automatic measurements. 

## 5. Conclusions

The overall findings of the study provide a validated, transparent, and reliable AI-based algorithm for the automated assessment of lower limb alignment on weight-bearing FLR, with a clear distinction between pre- and postoperative analysis. The AI algorithm is able to determine five key lower limb alignment angles with excellent reliability and accuracy. Based on these results, we propose the two main following use cases. First, the algorithm may be used to independently analyze large datasets for research purposes. Secondly, it could be used in supervision to support clinicians in time-consuming manual routine measurements. 

## Figures and Tables

**Table 1:** Intra-rater reliability of the manual measurements; rater 2a vs rater 2b; preoperative *n* = 95, postoperative *n* = 105.

Intra-Rater Reliability (Rater 2a vs. Rater 2b)						
	Statistical Method	mMPTA [°]	mLDFA [°]	mFAmTA [°]	mLDTA [°]	FSAmTA [°]
Preoperative	ICC (95% CI)	0.97 (0.95–0.98)	0.95 (0.93–0.97)	1.0 (1.0–1.0)	0.97 (0.96–0.98)	1.0 (1.0–1.0)
	Mean error (95% CI)	−0.07 (−0.27–0.13)	−0.02 (−0.22–0.19)	−0.01 (−0.06–0.05)	−0.13 (−0.37–0.12)	−0.05 (−0.14–0.03)
	SD	0.97	1.0	0.26	1.19	0.4
	RMSE	0.97	1.0	0.26	1.2	0.41
	Pearson correlation r (*p*-value)	0.97 (<0.001)	0.95 (<0.001)	1.0 (<0.001)	0.97 (<0.001)	1.0 (<0.001)
Postoperative	ICC (95% CI)	0.96 (0.94–0.97)	0.97 (0.95–0.98)	1.0 (0.99–1.0)	0.96 (0.94–0.97)	0.99 (0.99–1.0)
	Mean error (95% CI)	0.0 (−0.1–0.09)	−0.05 (−0.2–0.1)	−0.01 (−0.07–0.04)	0.15 (−0.1–0.4)	0.01 (−0.06–0.08)
	SD	0.48	0.77	0.28	1.29	0.37
	RMSE	0.48	0.77	0.28	1.3	0.37
	Pearson correlation r (*p*-value)	0.96 (<0.001)	0.97 (<0.001)	1.0 (<0.001)	0.96 (<0.001)	1.0 (<0.001)

ICC, intraclass correlation coefficient; CI, confidence interval; SD, standard deviation; RMSE, root mean square error.

**Table 2:** Inter-rater reliability of the preoperative manual measurements; rater 1 vs rater 2a, rater 1 vs. rater 2b; *n* = 95.

Inter-Rater Reliability (Rater 1 vs. Rater 2a)						
	Statistical Method	mMPTA [°]	mLDFA [°]	mFAmTA [°]	mLDTA [°]	FSAmTA [°]
Preoperative	ICC (95% CI)	0.91 (0.87–0.94)	0.91 (0.87–0.94)	0.99 (0.99–0.99)	0.88 (0.82–0.92)	0.99 (0.99–0.99)
	Mean error (95% CI)	0.18 (−0.14–0.49)	0.07 (−0.19–0.33)	0.09 (−0.14–0.32)	−0.46 (−0.91–−0.01)	0.1 (−0.11–0.31)
	SD	1.53	1.29	1.11	2.0	1.03
	RMSE	1.55	1.29	1.11	2.0	1.03
	Pearson correlation r (*p*-value)	0.92 (<0.001)	0.91 (<0.001)	0.99 (<0.001)	0.89 (<0.001)	0.99 (<0.001)
Inter-rater Reliability (Rater 1 vs. Rater 2b)						
Preoperative	ICC (95% CI)	0.89 (0.84–0.93)	0.92 (0.88–0.95)	0.99 (0.99–0.99)	0.85 (0.78–0.90)	0.99 (0.99–0.99)
	Mean error (95% CI)	0.11 (−0.24–0.45)	0.05 (−0.2–0.31)	0.08 (−0.14–0.31)	−0.59 (−1.09–−0.09)	0.05 (−0.16–0.26)
	SD	1.69	1.26	1.09	2.43	1.05
	RMSE	1.7	1.26	1.1	2.5	1.05
	Pearson correlation r (*p*-value)	0.89 (<0.001)	0.92 (<0.001)	0.99 (<0.001)	0.87 (<0.001)	0.99 (<0.001)

ICC, intraclass correlation coefficient; CI, confidence interval; SD, standard deviation; RMSE, root mean square error.

**Table 3:** Inter-rater reliability between AI method and manual measurements; preoperative *n* = 94 for mMPTA, mLDTA, FSAmTA, *n* = 88 for mLDFA, mFAmTA; postoperative *n* = 102 for mMPTA, mLDTA, *n* = 100 for FSAmTA, *n* = 99 for mLDFA, *n* = 97 for mFAmTA.

Inter-Rater Reliability (AI method vs. Rater 1)						
	Statistical Method	mMPTA [°]	mLDFA [°]	mFAmTA [°]	mLDTA [°]	FSAmTA [°]
Preoperative	ICC (95% CI)	0.86 (0.80–0.91)	0.84 (0.76–0.89)	1.0 (0.99–1.0)	0.88 (0.77–0.93)	0.99 (0.98–0.99)
	Mean error (95% CI)	−0.29 (−0.66–0.08)	0.15 (−0.26–0.56)	−0.03 (−0.17–0.11)	0.97 (0.56–1.38)	0.24 (0.0–0.48)
	SD	1.8	1.92	0.67	1.98	1.15
	RMSE	1.83	1.93	0.67	2.2	1.18
	Pearson correlation r (*p*-value)	0.87 (<0.001)	0.84 (<0.001)	1.0 (<0.001)	0.90 (<0.001)	0.99 (<0.001)
Inter-rater Reliability (AI method vs. Rater 2a)						
Preoperative	ICC (95% CI)	0.90 (0.85–0.93)	0.80 (0.71–0.87)	0.99 (0.99–1.0)	0.95 (0.92–0.97)	0.99 (0.98–0.99)
	Mean error (95% CI)	−0.19 (−0.53–0.14)	0.19 (−0.27–0.65)	0.0 (−0.19–0.19)	0.49 (0.21–0.78)	0.26 (0.02–0.49)
	SD	1.61	2.16	0.89	1.38	1.14
	RMSE	1.62	2.17	0.89	1.46	1.16
	Pearson correlation r (*p*-value)	0.91 (<0.001)	0.81 (<0.001)	0.99 (<0.001)	0.96 (<0.001)	0.99 (<0.001)
Postoperative	ICC (95% CI)	0.83 (0.76–0.88)	0.87 (0.81–0.91)	0.99 (0.98–0.99)	0.94 (0.91–0.96)	0.99 (0.98–0.99)
	Mean error (95% CI)	−0.13 (−0.32–0.07)	−0.31 (−0.63–0.01)	0.15 (0.05–0.24)	0.23 (−0.06–0.52)	−0.07 (−0.17–0.04)
	SD	1.0	1.61	0.48	1.47	0.53
	RMSE	1.0	1.64	0.51	1.49	0.53
	Pearson correlation r (*p*-value)	0.83 (<0.001)	0.88 (<0.001)	0.99 (<0.001)	0.94 (<0.001)	0.99 (<0.001)
Inter-rater Reliability (AI method vs. Rater 2b)						
Preoperative	ICC (95% CI)	0.88 (0.83–0.92)	0.83 (0.75–0.88)	0.99 (0.99–1.0)	0.95 (0.92–0.97)	0.99 (0.98–0.99)
	Mean error (95% CI)	−0.26 (−0.61–0.08)	0.19 (−0.24–0.62)	−0.01 (−0.18–0.17)	0.35 (0.04–0.65)	0.2 (−0.02–0.43)
	SD	1.68	2.02	0.82	1.49	1.11
	RMSE	1.7	2.03	0.82	1.53	1.13
	Pearson correlation r (*p*-value)	0.89 (<0.001)	0.83 (<0.001)	0.99 (<0.001)	0.95 (<0.001)	0.99 (<0.001)
Postoperative	ICC (95% CI)	0.85 (0.78–0.89)	0.85 (0.79–0.90)	0.99 (0.98–0.99)	0.95 (0.92–0.96)	0.99 (0.98–0.99)
	Mean error (95% CI)	−0.14 (−0.33–0.04)	−0.39 (−0.73–−0.06)	0.16 (0.07–0.25)	0.37 (0.09–0.65)	−0.08 (−0.2–0.03)
	SD	0.94	1.65	0.43	1.42	0.57
	RMSE	0.95	1.7	0.46	1.46	0.58
	Pearson correlation r (*p*-value)	0.85 (<0.001)	0.87 (<0.001)	0.99 (<0.001)	0.95 (<0.001)	0.99 (<0.001)

ICC, intraclass correlation coefficient; CI, confidence interval; SD, standard deviation; RMSE, root mean square error.

## Data Availability

Not applicable.
